# Endoscopic hand suturing after advanced endoscopic procedures: early outcomes of 31 cases in the upper and lower gastrointestinal tract (with video)

**DOI:** 10.20452/wiitm.2025.17928

**Published:** 2025-02-10

**Authors:** Zofia Orzeszko, Przemysław Kasprzyk, Urszula Zawada, Mirosław Szura, Michał Spychalski

**Affiliations:** Department of Surgery, Faculty of Health Sciences, Jagiellonian University Medical College, Kraków, Poland; Department of General and Oncological Surgery, Hospital of Brothers Hospitallers of St. John of God, Kraków, Poland; Center of Bowel Treatment, Brzeziny, Poland; Department of General and Oncological Surgery, Medical University of Lodz, Łódź, Poland

**Keywords:** complication management, endoscopic closure techniques, endoscopic hand suturing, endoscopic submucosal dissection, third

## Abstract

**INTRODUCTION:**

Endoscopic hand suturing (EHS) has emerged as a promising modality in gastrointestinal (GI) endoscopic procedures. Reports on its effectiveness in clinical practice remain limited due to its recent adoption.

**AIM:**

This study aimed to describe a single­ center experience regarding EHS and its outcomes.

**MATERIALS AND METHODS:**

This single ­center retrospective study analyzed individuals that underwent advanced endoscopic procedures in the upper and lower GI tract followed by EHS. Defined features (suturing time and speed) and outcomes (postprocedural bleeding, abdominal pain) were assessed.

**RESULTS:**

Thirty­ one patients were included in the analysis. The median (interquartile range [IQR]) size of the resected lesions was 20 (20–30) mm, and the median (IQR) diameter of the sutured defects was 25 (20–31) mm. The overall suturing time was 25 minutes, with a mean (SD) speed of 1.12 (0.5) mm/min. It varied in different locations, with the fastest closure in the proximal stomach (mean [SD], 25 [13.1] min; 1.27 [0.32] mm/min) and the longest in the rectum (mean [SD], 33 [16.2] min; 0.92 [0.4] mm/min). No symptoms of GI bleeding were reported during early and 4­week follow­up. One case (4.5%) of abdominal pain was reported for the upper GI tract, and none for the lower GI tract.

**CONCLUSIONS:**

EHS is a safe and effective technique for managing defects in both gastric and rectal advanced endoscopic procedures. Its potential application in preventing post­endoscopic submucosal dissection bleeding in high­risk patients is promising. The duration and complexity of the procedure remain the challenges that may limit its broader adoption. Further research and standardized training are imperative to optimize EHS outcomes and establish it as a routine practice in endoscopic surgery.

## INTRODUCTION

Third‑space endoscopy procedures have significantly developed in the past 2 decades. The techniques such as endoscopic sub‑ mucosal dissection (ESD), peroral endoscopic myotomy (POEM), submucosal tunnelling endoscopic resection (STER), and endoscopic full‑thickness resection (EFTR) have entered the routine practice in Europe and the United States, following their expansion in Eastern Asia. The indispensable role of ESD in the modern treatment of gastrointestinal (GI) cancer is highlighted by the latest recommendations from the European Society for Gastrointestinal Endoscopy^1^ and the Japan Gastrointestinal Endoscopy Society.^2^^,^^3^ Although these procedures are minimally invasive, they can be associated with various complications depending on the location, size, and histology of the lesion, as well as the patient’s overall condition.

The most common complications include perforation, immediate bleeding, and delayed bleeding. Perforations are usually diagnosed and treated during the primary procedure. The median (interquartile range [IQR]) incidence of perforation is greater following ESD (7.29% [6%–8.7%]) than endoscopic mucosal resection (EMR; 1.1% [0.1%–2.2%]). Immediate bleeding is commonly associated with advanced endoscopic resections and requires standard interventions. The intra‑ procedural bleeding rate was estimated at 10%.^4^ On the other hand, delayed bleeding could potentially manifest up to 4 weeks after the procedure. The median (IQR) delayed bleeding rate is similar for EMR and ESD at 2.2% (1.5%–3%) and 4% (3.5%–4.5%), respectively.^4^ Notably, employing endoscopic clip closure for mucosal defects after the resection of large colon polyps (>20 mm) may reduce the risk of postpolypectomy bleeding from 7.1% in the control group to 3.5% in the clip group.^5^ However, prophylactic clipping of smaller polyps has not been shown to reduce the risk.6 A novel method of endoscopic hand suturing (EHS) has a potential to mitigate the risk of delayed bleeding,^7^^,^^8^^,^^9^ improve the healing process,^10^ and effectively close a full‑wall defect after endoscopic resection. EHS has emerged as a promising modality in endoscopic procedures, particularly after ESD. Recent studies have assessed the feasibility, safety, and efficacy of EHS, demonstrating its potential in preventing postprocedural complications.^7^^,^^8^^,^^9^^,^^11^ However, reports on its effective‑ ness in clinical practice remain limited due to its recent adoption.

## AIM

This study outlines our experience integrating EHS into regular clinical practice following endoscopic resections in the upper and lower GI tract. The implementation of the technique was carried out at a specialized facility routinely performing advanced third‑space procedures, including ESD, POEM, and STER. The study aimed to describe the single‑center experience of the first 31 cases of EHS and investigated the technique and its outcomes. We were especially interested in evaluating the safety of the endoscopic suturing and the prevalence of complications following the procedure.

## MATERIALS AND METHODS

This single‑center retrospective study analyzed individuals who underwent advanced endoscopic procedures followed by EHS from March 2023 to June 2024. The procedures were conducted both in the up‑ per and lower GI tract and included endoscopic intermuscular dissection (EID), EMR, ESD, EFTR, POEM, and STER. All procedures were performed in a conventional manner. EHS was performed to close a GI wall defect (after EMR, ESD, or EID) or a linear entry mucosal incision (after POEM or STER). The indications for additional suturing included a high risk of bleeding following ESD in the upper GI tract (based on the patient history of anticoagulant therapy and advanced age), closing a deep wall defect after EID in the rectum to improve recovery, closing a full‑thickness defect after EFTR in the cases of submucosal lesions, and concurrent treatment for perforation during ESD. The primary outcomes were suturing time and speed. The suturing time was defined as the time between delivering and retrieving the needle, based on video recordings and measured in minutes. The suturing speed was calculated by dividing the length of the defect in millimeters by the suturing time in minutes. The length of the defect was assessed by an endoscopist and then confirmed by measuring the retrieved specimen. Postprocedural bleeding rate was considered a secondary outcome.

All procedures were performed by a single experienced surgeon‑endoscopist,^12^ accompanied by a qualified assistant (nurse). The implementation of the EHS was preceded by ex vivo training. We recorded the first procedures performed and obtained a positive evaluation from professor Osamu Goto, an inventor of this method.^13^

The patients were admitted 1 day before the procedure and prepared conventionally. Antithrombotic agents were managed according to recommendations of the British Society of Gastroenterology and the European Society of Gastrointestinal Endoscopy.^14^

### Suturing technique 

EHS was conducted using a set of equipment and suturing techniques (see Video). The following equipment was used: flexible scopes (EG29‑I20C 3.2 mm channel, EC34‑I10cF3.8 mm channel, EC34‑I10cF 3.8 mm channel, all Pentax, Tokyo, Japan), an endoscopic needle‑holder (EHSFG‑260 Olympus, Tokyo, Japan), and sutures (VLOC L0604, absorbable barbed suture, Covidien, Mansfield, Massachusetts, United States). The distal attachment was used for optimal view and additional assistance. A suture was primarily triple knotted at the distal end, ex vivo. After placing the needle‑holder in the scope channel, the needle was placed in the needle‑holder and inside the distal attachment, then thoroughly delivered to the suturing site with a scope. A defect was closed with a continuous suture, starting on the anal side, in the anterograde position. The suturing method was based on the surgical approach. For this technique, the needle was held within the needle holder on the opposite side from the tip. The needle was pierced perpendicularly into the tissue at the side of the wound with an appropriate margin, then driven through the tissue with rotation and grasped at the bot‑ tom of the defect. The same steps were repeated from the middle of the wound to create a symmetrical structure. The thread was tightened after each stitch to close the margins of the defect. The depth of the suture was adjusted to the procedure. The full‑thickness sutures were applied after full‑thickness resections and in the case of iatrogenic perforation during ESD within the up‑ per GI tract. After a complete closure, the suture was cut with endoscopic scissors (single‑use loop cutter, FS‑410U Olympus, Tokyo, Japan) and re‑ moved in the distal attachment.

**TABLE 1:** Clinical characteristics of the procedures (n = 31)

Parameter		Value
Women, %		
Age, y, mean (SD)		
Prior biopsy, %		
Lesion location, n (%)	Rectum	9 (29)
	Proximal stomach	10 (32.3)
	Distal stomach	10 (32.3)
	Esophagus	1 (3.2)
	Duodenum	1 (3.2)
Morphology, Paris classification, n (%)	0­‑Ip	1 (3.2)
	0­‑Is	5 (16.1)
	0­‑IIa+c	7 (22.6)
	LST­‑G	3 (9.7)
	LST­‑NG	1 (3.2)
	Not applicable	14 (45.2)
Type of procedure, n (%)	EMR	1 (3.2)
	ESD	16 (51.6)
	EID	6 (19.4)
	EFTR	5 (16.1)
	STER	2 (6.5)
	POEM	1 (3.2)

Abbreviations: 0­Ip, pedunculated polyp; 0­Is, sessile polyp; 0­IIa+c, flat elevated polyp with central depression; EFTR, endoscopic full­thickness resection; EID, endoscopic intermuscular dissection; EMR, endoscopic mucosal resection; ESD, endoscopic submucosal dissection; LST­G, laterally spreading tumor granular type; LST­NG, laterally spreading tumor nongranular type; POEM, peroral endoscopic myotomy;

**TABLE 2:** Clinical characteristics of the procedures (n = 31)

Parameter	Value
Antithrombotic agent intake	7 (22.6)
Oral antidiabetics intake	5 (16.1)
Lesion size, mm	20 (20–30)
Specimen (defect) size, mm	25 (20–31)
EFTR with peritoneal access	5 (16)
Overall procedure time, min, mean (SD)	60 (21)
En­‑bloc resection	31 (100)
Median length of stay, d	2 (1–3)

Data are presented as number (percentage) or median (interquartile range) unless provided otherwise.

Abbreviations: see TABLE 1

### Postprocedural management 

After gastric procedures, a proton pump inhibitor was administered orally for 6 weeks. The patients were allowed to drink water on postoperative day 1 (POD1) and stayed on a clear liquid diet for 7 days, when the solid diet was allowed. The patients were dis‑ charged on POD2 after the upper GI tract procedure and on POD1 after the lower GI tract procedure. The first visit at an outpatient clinic was scheduled 4 weeks after the procedure to follow up on any postdischarge events. Follow‑up was based on comprehensive clinical evaluation with‑ out second‑look endoscopy.

### Statistical analysis

Statistical analyses were performed using SPSS 29.0.2.0 software for MacOS (SPSS Inc., Chicago, Illinois, United States). Descriptive statistical methods were used for the analysis. Continuous variables were presented as mean and SD or median and IQR, depending on the normality of the data distribution.

### Ethics

The study was approved by the Institutional Review Board (118.0043.1.330.2024) and registered at ClinicalTrials.gov (NCT06622746). As all analyses were based on a review of data‑ base records, the study did not require additional consents.

## RESULTS

**FIGURE 1:**
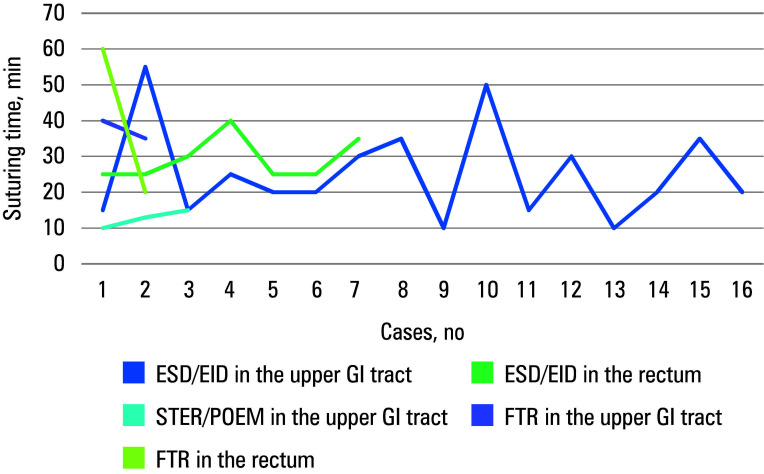
Learning curve of the suturing time

**FIGURE 2:**
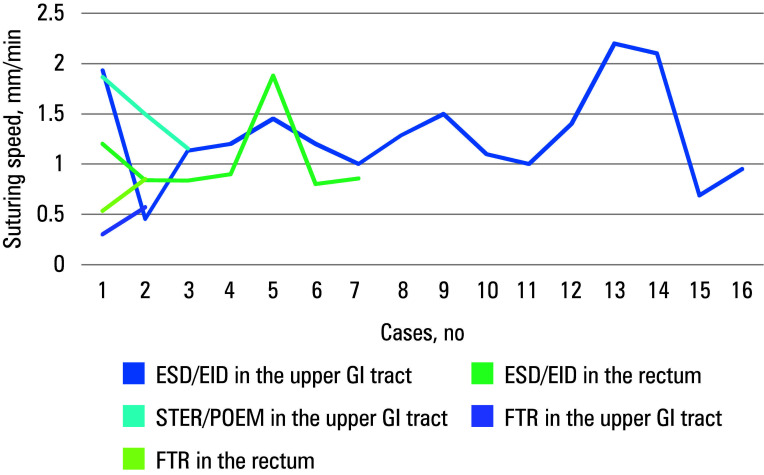
Learning curve of the suturing speed

Thirty‑one patients were included in the analysis. All of them underwent advanced endoscopic resections followed by defect closure using EHS. The study population and the lesions are described in TABLE 1. Mean (SD) age of the participants was 66.5 (10.7) years, and 52% were women. Seven participants (22.6%) were treated with antithrombotic agents (all with direct oral anticoagulants), and 5 patients (16.1%) took oral antidiabetic agents. All closures were complete TABLE 2. Nine procedures were performed in the rectum, 10 in the proximal stomach, 10 in the distal stomach, 1 in the esophagus, and 1 in the duodenum. They included 16 cases of ESD, 6 EIDs, 5 EFTRs, 2 STERs, 1 POEM, and 1 en‑bloc EMR. The lesions were of various morphology TABLE 1. Polyps were mostly (22.6%) described as 0‑IIa+c according to the Paris classification.15 The median (IQR) size of the resected lesions was 20 mm (20–30 mm). The median (IQR) diameter of the sutured defect, corresponding to a specimen size, was 25 (20–31) mm TABLE 2. Five procedures were considered EFTRs TABLE 2. Two cases of EFTR were due to iatrogenic perforation identified during ESD, and were managed immediately with full‑thickness EHS. These patients required prolonged hospital stay (discharge on POD5 and POD7) and additional antibiotic course. A complete defect closure was achieved in both cases, and no postprocedural complications were observed on follow‑up. The case of duodenal perforation suturing was previously described.16 Other procedures were performed without intraoperative complications. The mean (SD) procedure time was 60 (21) minutes with a median (IQR) suturing time of 25 (19–35) minutes. En‑bloc resections were achieved in all cases. The median (IQR) length of hospital stay was 2 (1–3) days TABLE 2. The suturing time and speed in different locations are presented in TABLE 3 . The median (IQR) overall suturing time was 25 (19–35) minutes, at a mean (SD) speed of 1.12 (0.5) mm/min. It varied at different locations, with the fastest closure in the proximal stomach (mean [SD], 25 [13.1] min; 1.27 [0.32] mm/min) and the longest in the rectum (mean [SD], 33 [16.2] min; 0.92 [0.4] mm/min). The learning curves are presented separately for the suturing time FIGURE 1 and speed FIGURE 2.

**TABLE 3:** Suturing time and speed in different locations (n = 30)^a^

Parameter		Value
Suturing time, min	Overall	25 (19–35)
	Proximal stomach	25 (13.1)
	Distal stomach	25.5 (13.8)
	Rectum	33 (16.2)
Suturing speed, mm/min	Overall	1.12 (0.5)
	Proximal stomach	1.27 (0.32)
	Distal stomach	1.22 (0.69)
	Rectum	0.92 (0.4)

Data are presented as mean (SD) or median (interquartile range).

a Suturing in the duodenum was excluded.

**TABLE 4:** Early postprocedural course in the study group (n = 31)

Parameter		Upper GI tract procedures (n = 22)	Lower GI tract procedures (n = 9)
GI bleeding		0	0
Abdominal pain		1 (4.5)	0
Oral nutrition status on POD1	Restricted	4 (18.2)	0
	Still water	7 (31.8)	8 (88.9)
	Liquid diet	11 (50)	1 (11.1)
Solid food intake, POD, d		7 (7–8)	–

Data are presented as number (percentage) or median (interquartile range).

Abbreviations: GI, gastrointestinal; POD, postoperative day

Four dissections in the stomach and 5 in the rectum were followed by a histological diagnosis of adenocarcinoma, with complete resection (R0) in all cases. In 2 rectal cases treated with EID, the cancer invaded the muscle layer (T2 in the Tumor‑Node‑Metastasis [TNM] classification), and the patients were referred for surgery. In other cases, the invasion was limited to the submucosal layer (T1 in the TNM classification), and all the patients were referred for oncological surveillance.

The postprocedural course was assessed separately for the upper and lower GI tract TABLE 4. For the upper GI tract, there was 1 case of abdominal pain (4.5%) located in the left lower quadrant, and it was treated conservatively. No complaints were reported following the lower GI tract surgeries. Overall, no symptoms of GI bleeding occurred after EHS closure, either in the upper or lower GI tract, in the early postprocedural period and on 4‑week follow‑up. The oral nutrition on POD1 varied TABLE 4. It was completely restricted after 4 upper GI tract procedures (18.2%), while the patients were allowed to drink still water in 7 cases (31.8%), and to follow liquid diet in 11 cases (50%). After the lower GI tract procedures, 8 patients (88.9%) were allowed to drink still water on POD1, while in 1 case a liquid diet was allowed (11.1%). A light solid diet was allowed on POD7 and was well tolerated, with no complaints.

## DISCUSSION 

EHS represents an innovative technique for closing resection sites, especially after ESD. Previous studies have assessed the feasibility, safety, and efficacy of EHS, demonstrating its potential in preventing postprocedural complications. This study explored the technique and its clinical outcomes following gastric and colorectal endoscopic procedures.

Recent findings have supported EHS as a valuable technique in preventing bleeding following gastric ESD in high‑risk patients, particularly those on antithrombotic therapy. Our results support the existing evidence on the safety and efficiency of EHS in preventing gastrointestinal bleeding in participants with different antithrombotic status. No symptoms of bleeding were documented on follow‑up. Accordingly, Goto et al^7^ conducted a prospective multicenter study demonstrating the feasibility and safety of EHS following gastric ESD in patients continuously treated with antithrombotic agents. The study found that 83% of the suture lines were well‑maintained on POD3, with only 10% of cases experiencing major bleeding requiring emergency intervention, and the subclinical bleeding rate of 2%, indicating that EHS could be a valuable option for man‑ aging high‑risk patients. The impact of EHS on the patients taking antithrombotic agents was also investigated by Akimoto et al.8 In a single‑‑arm prospective trial involving 20 patients, no postprocedural bleeding was observed. The median (IQR) number of stiches was 6 (4–8), and the suturing time was 36 (24–60) minutes. All sutures remained intact during early postoperative surveillance, with no adverse events. These findings support EHS as a valuable technique in preventing bleeding after gastric ESD in high‑‑risk patients, particularly those on antithrombotic therapy.

Furthermore, ESD is recommended for the treatment of early rectal cancer.^2^^,^^3^ Previous studies showed similar efficiency of ESD and transanal endoscopic microsurgery in achieving R0 resection; however, the safety of this procedure varied in different reports.^17^^,^^18^ EHS may be efficiently implemented in the colorectal setting to prevent early complications. Our findings demonstrated the safety and effectiveness of this approach, as confirmed by recent publications. Uozumi et al^9^ assessed 20 cases where colorectal ESD was followed by EHS using a modified, flexible, through‑the‑scope needle holder. They included neoplasms of a median (IQR) size 37 (21–65) mm located in the sigmoid colon and the rectum. A complete closure was achieved in 90% of cases, with a sustained closure rate of 85% on POD3. The median (IQR) suturing time was 49 (23–92) minutes. The absence of adverse events further supported the safety of EHS in a colorectal setting. The authors pointed out a relevant role of EHS in the cases with a high risk of delayed complications. Furthermore, a pilot study by Abe et al^11^ evaluated EHS in colorectal ESD, reporting a complete closure rate of 73% and a sustained closure rate of 64% by POD3 or POD4. The median (IQR) number of stitches was 8 (6–12). The postprocedural bleeding occurred in 9% of cases. The authors demonstrated that despite the median (IQR) suturing duration of approximately 56 (30–120) minutes, EHS may provide a complete and sustained closure of colorectal defects. In this study, we achieved a 100% closure rate, surpassing the rates reported in the referenced studies. However, if the postprocedural course was uneventful, no second‑look endoscopy was performed to assess its sustainability as in common clinical practice.

Change Image

Abbreviations: see TABLE 1 and TABLE 4

Change Image

Abbreviations: see TABLE 1 and TABLE 4

A few articles have discussed extended suturing time in endoscopic procedures. In comparison with the literature reports,^8^^,^^9^^,^^11^ our study demonstrated a significantly shorter suturing time. The learning curve for the rectal suturing time showed insignificant changes for submucosal defects in 7 cases. In the upper GI tract, the duration of the procedures varied. More cases of full thickness suturing are required for definite conclusions. Despite the suturing time being directly influenced by the size of the defect, we standardized the results by calculating the suturing speed. This revealed the lowest speed in the rectum, intermediate in the distal stomach, and the highest in the proximal stomach. The learning curve indicated a consistent speed among 7 rectal cases and 16 upper GI tract cases, raising questions about the number of cases needed to achieve proficiency. Scheppach et al^19^ described an improvement in mean stitching time after 4 cases performed in a constant team; however, the mean suturing time was longer than described in this study, and a different device was used. The su‑ turing speed was not addressed in other publications. However, it was suggested that the learning curve may be further improved by technological advancements.^20^ It is imperative to acknowledge that the procedures we described were conducted by a proficient team of skilled professionals, and replicating their outcomes under different conditions may present significant challenges. Nevertheless, various training methods exist to facili‑ tate the development and enhancement of essential skills ex vivo.^21^ It is vital to assess the learning curve based on trainees in forthcoming trials. 

The potential application of EHS may be expanded to various indications. It was reported as safe and sufficient for closing the defects after gastric subepithelial lesion removal under laparoscopic guidance, rectal EFTR, thoracoscopy‑assisted esophageal EFTR, POEM, gastric POEM, antireflux mucoplasty, postoperative anastomotic leak, and mucosal closure for gastric ulcer bleeding.^22^^,^^23^^,^^24^^,^^25^^,^^27^ Moreover, it was successful for closing rare fistulas after a removal of a lumen‑apposing metal stent^28^ and preventing self‑expandable metal stents from migrating in the digestive tract.^29^ In addition, Okamura et al^30^ recently described the case of EHS following ESD at the anastomosis after right hemicolectomy. Finally, according to Liu et al,^31^ EHS was successfully used to treat elective full‑thickness duodenal resection as an alternative to clip closure and laparoscopic suturing. Therefore, we implemented EHS as a concurrent therapeutic solution in 2 cases of iatrogenic perforations in the upper GI tract. The procedures were successful, and the subsequent recovery was uneventful in both cases. Nonetheless, supplementary antibiotic treatment was administered, and hospital discharge was delayed until POD5 and POD7. It is important to note that these outcomes are casuistic and insufficient to advocate for a widespread application of this approach. A comprehensive investigation involving a larger sample size is imperative to further assess EHS safety and efficacy.

As the EHS has been recently developed, various aspects of this technique are currently under review. An alternative technique for delivering the needle to the suturing site using an overtube was outlined.^8^ We introduced a method involving placing the needle in the needle‑holder and inside the distant attachment. This approach proved to be safe and effective. The needle was extracted using the same method. Nonetheless, further investigation and comprehensive consideration of the techniques for delivering the needle to the site are in demand.

Previous reports described securing the last stitch with single or multiple clips.^8^ Innovative strategies combining EHS with clips have been developed to enhance closure and prevent com‑ plications after ESD and ESD with myotomy (ESD‑ME) in the rectum. This technique involves 2 steps, that is, endoscopic sewing of the resec‑ tion site and closing any remaining defects with the clips. The sustained closure on POD3 to POD5 was achieved in 73.8% of ESDs and 71.4% of ESD‑MEs. A margin of the stitches of at least 5 mm was identified as an independent factor for sustained closure, emphasizing the importance of precision in this technique.^32^ No delayed bleeding was observed. Instead, we performed all closures with sutures alone with satisfactory outcomes. The largest defect in our study mea‑ sured 55 mm, and it was closed completely, with no adverse events. However, the need for addi‑ tional clipping is still debatable and requires fur‑ ther research.

Long‑term outcomes and healing after EHS have also been explored. Higuchi et al^33^ noted that mucosal deformity induced by EHS dis‑ appeared within 16 months, and the study by Akimoto et al^10^ on porcine gastric models demonstrated that EHS promoted faster and more complete healing, as compared with unsutured controls. The sutured defects showed restored epithelium and muscularis mucosae within 14 days, with decreased neovascularization and submucosal fibroblast rates, indicating enhanced healing and reduced inflammation. A thorough examination of long‑term outcomes and safety of surveil‑lance or potential surgery is needed.

Recent advancements in technology have improved the efficacy of EHS. Three‑dimensional endoscopy may serve as an effective training tool, enhancing the learning curve and efficiency of EHS.^20^ Additionally, training sheets for EHS may further aid in standardizing and improving the technique.^21^ Our performance was preceded by ex vivo training to obtain optimal results and guarantee patient safety. Comprehensive training is crucial for adapting this technique to the common practice.

Our findings confirm the safety and efficacy of EHS. However, it is imperative to acknowledge certain limitations of the study. The evaluation was retrospective and based on clinical data from an experienced endoscopy center. The follow‑up period was restricted to 4 weeks, and consequently, the potential for long‑term complications cannot be entirely dismissed. Additionally, given that the procedures were conducted by the proficient endoscopist in the specialized referral center, it is cautioned that the data may not be universally applicable to the centers with less experience in this specific indication.

## CONCLUSIONS

EHS is a safe and effective technique for managing defects in both gastric and rectal advanced endoscopic procedures. Its potential application in preventing post‑ESD bleeding in high‑risk patients, particularly those undergoing antithrombotic therapy, is promising.

The duration and complexity of the procedure remain challenges that may limit its broader adoption, nonetheless, a proficient team has the ability to improve all these aspects. The suturing speed was the highest for the proximal stomach, inter‑ mediate for the distal stomach, and the lowest for the rectum.

Further research is required to optimize the needle delivery techniques and assess the suture line protection methods, including clipping. Advances in technology and innovative strategies may enhance the efficacy and adoption of EHS. Further research and standardized training are essential to optimize EHS outcomes and establish it as a routine practice in endoscopic surgery.

## VIDEO

The video file is available online at https://dx.doi.org/10.20452/ wiitm.2025.17928.
